# Effect of Belladonna in Immediately Arresting the Secretion of Milk

**Published:** 1857-01

**Authors:** 


*Effect of Belladonna in Immediately Arresting the Secretion of Milk.*

Dr. R. H. Goolden has communicated to the *Lancet* (Aug. 9th, 1856) the two following cases, which seem to show that belladonna possesses the power of arresting the secretion of milk.

E. J., aged 28, was admitted into Anne's Ward, St. Thomas's Hospital, with a small child at the breast four months old. At the time of her admission she had swelling and acute pain in both wrists, right elbow, both knees, and left ancle. The knee-joints were distented with synovia, and erythematous patches were on the skin of the knees, ankles, and wrists. She was bathed in perspiration, and the secretion of milk was abundant. According to the regulation of the hospital, the child was removed; indeed, from her helpless condition, it was necessary, considering the difficulty of attending to an infant in a ward with other patients. Soon after her admission she took eight grains of calomel and a grain and a half of opium, followed by a senna draught; and one scruple of nitrate of potassa, ten grains of bicarbonate of potassa, and half a drachm of spirit of nitric ether, in peppermint water, every four hours. The joints were covered with cotton wool.

On the following day, at two o'clock, I found she had been freely purged; the joints were in nearly the same state. She had had no sleep. The breasts had become tumid, hard, painful, knotty, and extremely tender. The superficial veins were distended. Some milk had been drawn, but the process was attended with great pain, and we could not listen to the heart's sounds on account of the tenderness.

A milk abscess, in complication with rheumatic fever, was of all things to be avoided, and unless the secretion could be at once arrested it appeared inevitable. In this strait I recollected that I had somewhere met with an observation (but I cannot remember whether it was in an English or foreign journal) that atropine applied externally to the breasts would dry up the milk; and, thinking it reasonable, I caused the areolæ of the breasts to be smeared with extract of belladonna, in the same way that it is used to dilate the pupil of the eye. I likewise ordered the addition of half a drachm doses of colchicum wine, knowing that whenever milch cows eat the meadow saffron in the pasture they immediately become dry; and though I have not much faith in colchicum as a remedy in rheumatic fever uncomplicated with gout, there could be no objection to its use, and it has the sanction of much higher authority than my own.

On my third visit, the following day, the first inquiry was about the breasts. They were all right. But was it the colchicum or belladonna that had relieved them? The extract was used before I left the ward; before the mixture was given the secretion of milk had been arrested and the breasts had become soft. The rest of the case has no further special interest. I will only state that there was no heart affection, and that the fever, though very severe while it lasted, was of short duration, and the patient left the hospital quite well in fourteen days.

The second case that occurred to me was uncomplicated with any disease, and as would usuallly fall under the care of the accoucheur rather than the physician.

A lady, the wife of a clergyman, was traveling with her husband, and, in order to accompany him, had weaned her baby (then seven months old.) Happening to be at Oxford at the commemoration festival, he came to me in great trouble, telling me that his wife had done a foolish thing in weaning the child, and that they were now arrested in their progress in consequence of the state of her breasts. They were tumid, very tender, painful, and hard, with large superficial veins, and the milk had been drawn with difficulty several times with temporary relief. I recommended the application of the extract of belladonna to the areolæ, desiring them to send for a medical practitioner if the inconvenience did not immediately subside, or unless she felt quite well. A few days brought me a letter, giving a very satisfactory account, and thanking me for what she was pleased to call my wonderful prescription. Within two hours she was perfectly relieved, the milk absorbed, and (what is very important) there was no fever or other inconvenience attending the sudden suppression of the milk; and, instead of taking the opening medicines I had prescribed for her, she continued her journey the next morning.

I have not been able to discover that the fact that belladonna is available for the purpose of arresting the milk secretion is at all generally known—certainly it was not to several accoucheurs in large practice of whom I have inquired. The fact is important if true, for then milk abscesses will become a matter of past history, and probably many diseases of the breast may be rendered less complicated by its use.

The two cases I have detailed are not sufficient to prove that it will always be either successful or safe, but they render it highly probable that it is so.—*American Journal.*

